# Supplementary material for: Adaptations to cursoriality and digit reduction in the forelimb of the African wild dog (Lycaon pictus)
Source: PeerJ. 2020 Sep 7;8:e9866. doi: 10.7717/peerj.9866 (PMC7482643; doi:10.7717/peerj.9866)
Supplement: Supplemental Information 1 [file peerj-08-9866-s001.docx]

**Supplementary Table 1. Smith et al.** Descriptions of forelimb ligamentous morphology of *L. pictus*.

**Scapular ligaments:**

***Ligamentum acromiolabra***: The scapula was partially disarticulated during necropsy with little ligamentous attachment of the shoulder girdle remaining. However, a ligamentous band courses from the craniomedial surface of the acromion transversely toward the glenoid. It attaches to the lateral border of the glenoid labrum and the bony craniolateral margin of the glenoid. This ligament separates the tendons of mm. supraspinatus and infraspinatus.

***Tendon of m. biceps brachii***: The tendon is robust and curving, 8.2 mm wide and 2.8 mm thick. It has a tight origin from the supraglenoid tubercle of the scapula, which is quite extensive in *L pictus.* The tendon is enveloped by a synovial sheath, and its course takes a broad arc over the glenohumeral joint before passing deep to the retinaculum transversum humerale. The tendon courses through the length of the muscle belly.

**Cubital ligaments:**

***Ligamentum collaterale cubiti mediale***: This is a V-shaped ligament in the medial cubital region consisting of two crura. The cranial crus is stout and cordlike, and originates from the medial epicondyle of the humerus. Its fibers run distally parallel to the orientation of the antebrachium to insert onto the radial tuberosity, proximal ulna, and interosseous membrane. The caudal crus originates from a ridge on the distal aspect of the medial condyle, caudal to the origin of the cranial crus. Its fibers fan out to insert broadly along the proximomedial ulna proximal to the coronoid.

***Ligamentum collaterale cubiti laterale***: This thick, fan-shaped ligament is the broadest of the cubital ligaments. It originates via a tight attachment to the lateral epicondyle of the humerus. It fans out to cover the lateral epicondyle and then divides into two crura. The cranial crus is oriented distally and inserts onto a small tubercle on the lateral aspect of the radial head and neck. The caudal crus courses caudodistally to insert onto the proximal ulna. The fibers of the cranial crus fuse with the lateral fibers of the ligamentum annulare radii. Unlike the domestic dog, there is no evidence of a sesamoid within this ligament.

***Ligamentum anulare radii***: This thin, band-like ligament wraps around the head of the radius. It attaches to the medial and lateral aspects of the coronoid process of the ulna. Its fibers fuse laterally with the cranial crus of the ligamentum collaterale cubiti laterale. Medially, it dives deep to the ligamentum collaterale cubiti mediale, but does not fuse with it. Its fibers are extremely thin, and minimal rotatory movement is possible at the radiohumeral joint.

***Oblique ligament***: This ligament was damaged during necropsy. However, one of its distal attachments on the radius was preserved. It is a thin slip of ligament attaching onto the ligamentum annulare radii at its mediolateral midpoint. Any other attachments that it may have had during life were not preserved after necropsy.

***Ligamentum olecrani***: This ligament was disconnected distally during necropsy, but its proximal portion was intact. It takes a broad origin from the deep medial margin of the olecranon fossa of the humerus. Its origin is much wider than in the domestic dog. Its fibers are extremely elastic and permits a great deal of mobility. It crosses the elbow caudally, but its insertion point on the craniomedial olecranon could not be determined due to damage.

**Carpal ligaments:**

***Ligamentum collaterale carpi mediale***: This medial ligament of the carpus consists of two distinct components, separated by the tendon of m. abductor digiti I longus. The cranial crus originates from a broad attachment on the distal 3 cm of the radial shaft. It spreads out to cross the carpus dorsomedially and attaches to the dorsal surfaces of the scapholunate (intermedioradial carpal), trapezoid (carpal II), and metacarpal II. This attachment is much more distally extending than in the domestic dog. The caudal crus originates from a tubercle on the distal radius proximal to the styloid process. It inserts broadly to the medial aspect of the scapholunate. Its lateral border contains a deep groove for passage of the tendon of m. abductor digiti I longus.

***Ligamentum collaterale carpi laterale***: This ligament is notably less extensive than its counterpart on the medial side, being both shorter and narrower. However, it is broader than that of the domestic dog. It attaches to the distal ulna along its lateral border, and then crosses the ulnocarpal joint to insert onto the triquetrum (ulnar carpal).

***Ligamentum radiocarpeum dorsale***: This ligament courses dorsally over the carpus. Proximally it attaches to the distal 2.5 cm of the radius. It thins out and crosses the radiocarpal joint dorsally before attaching to the triquetrum (ulnar carpal) and lateral aspect of the scapholunate (intermedioradial carpal).

***Ligamentum accessoriometacarpeum (pisimetacarpeum) IV***: This stout ligament is similar in morphology to its counterpart to digit V. It attaches to the palmodistal surface of the pisiform medial to the attachment of the ligament to digit V. It courses distally to attach to the base of metacarpal IV.

***Ligamentum radiocarpeum palmare***: This thin ligament originates from the palmar surface of the distal radius. It crosses the carpus obliquely laterally to insert onto the palmar surface of the scapholunate (intermedioradial carpal), and via a small slip to the pisiform.

***Ligamentum ulnocarpeum palmare***: This ligament is extremely small and attaches to the internal palmar surface of the distal ulna. Its fibers course almost transversely to attach via a very small connection to the pisiform immediately adjacent to the attachment of the ligamentum radiocarpeum palmare.

***Ligamentum intercarpea dorsalia (carpi transversum***, Davis, 1964): This is a thick, triangular ligament covering the palmar surface of the carpus. Proximally, it attaches broadly to the distal radius and ligamentum collaterale carpi mediale. Its fibers course distolaterally across the carpal joint to attach onto the palmar surfaces of the scapholunate and the medial surface of the pisiform. Its distalmost fibers continue on to attach onto the palmar surfaces of carpals II-V. Its palmar surface is concave to accommodate the passage of the tendons of m. flexor digitorum profundus.

***Ligamenta carpometacarpea palmaria***: These slender elongated ligaments support the joints between the distal carpal row and the metacarpals. Each ligament arises from the distal palmar aspect of a carpal, crosses the joint palmarly, and courses along the midline of the metacarpal shaft. They insert onto the palmar surfaces of the metacarpals. Lengths: II- 15,3 mm, III- 19.5 mm, IV- 26.5, V- 16.9. The ligaments to digits IV and V are the most robust, with IV being the largest. The ligament to digit IV was initially mistaken for a muscle, because it is so large.

***Ligamentum carpometacarpea dorsalia***: These short ligaments connect the distal carpal row with the bases of the metacarpals on the dorsal surface of the manus. They are quite small and relatively non-descript.

***Ligamenta metacarpea dorsalia et palmaria***: Proximally, the metacarpals are tightly adhered to each other making direct observation of these ligaments difficult. However, they appear to extend between adjacent metacarpal shafts for the proximal 25-30 mm.

**Metacarpophalangeal ligaments:**

***Ligamenta collaterale metacarpophalangea laterale et mediale***: Each digit is supported on either side of the metacarpophalangeal joint by medial and lateral ligamentum collaterale metacarpophalangea.

***Ligamenta palmaria***: These ligaments connect the medial and lateral sesamoids of each metacarpophalangeal joint. Their distinct fibrous bands course transversely and are concave to permit passage of the long flexor tendons. In the domestic dog, ligamenta sesamoidea cruciata are described as coursing between the bases of the sesamoids and the proximal phalanges. This morphology is not observed in *L. pictus.* Instead, the ligamenta palmaria extend further distally to attach firmly across the palmar surface of the base of the proximal phalanx.

***Ligamenta sesamoidea collateralia laterale et mediale***: These ligaments connect the sesamoids of each metacarpophalangeal joint to the proximal phalanx on either side. Distally their fibers converge with the corresponding ligamentum collaterale metacarpophalangeal on that side of the joint.

***Ligamentum anulare palmaris***: The palmar annular ligaments are composed of thickenings of the palmar fascia and digital fascia. They support the metacarpophalangeal and interphalangeal joints by wrapping circumferentially around them. Their fibers fuse with the accompanying fascia such that their annular bands are not clearly distinguishable from the adjacent connective tissue.

**Interphalangeal ligaments:**

***Ligamenta collaterale PIP mediale et laterale***: Each proximal interphalangeal joint is supported medially and laterally by collateral ligaments. The ligaments course from the side of the head of the proximal phalanx to the base of the intermediate phalanx.

***Ligamenta collaterale DIP mediale et laterale***: Each distal interphalangeal joint is similarly supported medially and laterally by collateral ligaments. The ligaments course from the side of the head of the intermediate phalanx to the base of the distal phalanx.

***Ligamenta dorsalia*:** The dorsal elastic ligaments course along the dorsal surface of each intermediate phalanx. The ligaments start proximally just distal to the base of the intermediate phalanx on the side towards the middle of the pes. The ligaments to digits II and III are therefore positioned medially on the middle phalanx, while the ligaments to digits IV and V are positioned laterally. Unlike the domestic dog, each digit possesses only one robust dorsal elastic ligament, rather than two smaller such ligaments that converge. The ligament crosses the distal interphalangeal joint to insert onto the dorsal surface of the base of the distal phalanx. As the name suggests, the tissue is quite elastic and responsive. From largest and most robust to smallest and most gracile: 2 > 5 > 4 > 3.
