# Supplementary figures and images for: Adaptations to cursoriality and digit reduction in the forelimb of the African wild dog (Lycaon pictus)
Source: PeerJ. 2020 Sep 7;8:e9866. doi: 10.7717/peerj.9866 (PMC7482643; doi:10.7717/peerj.9866)

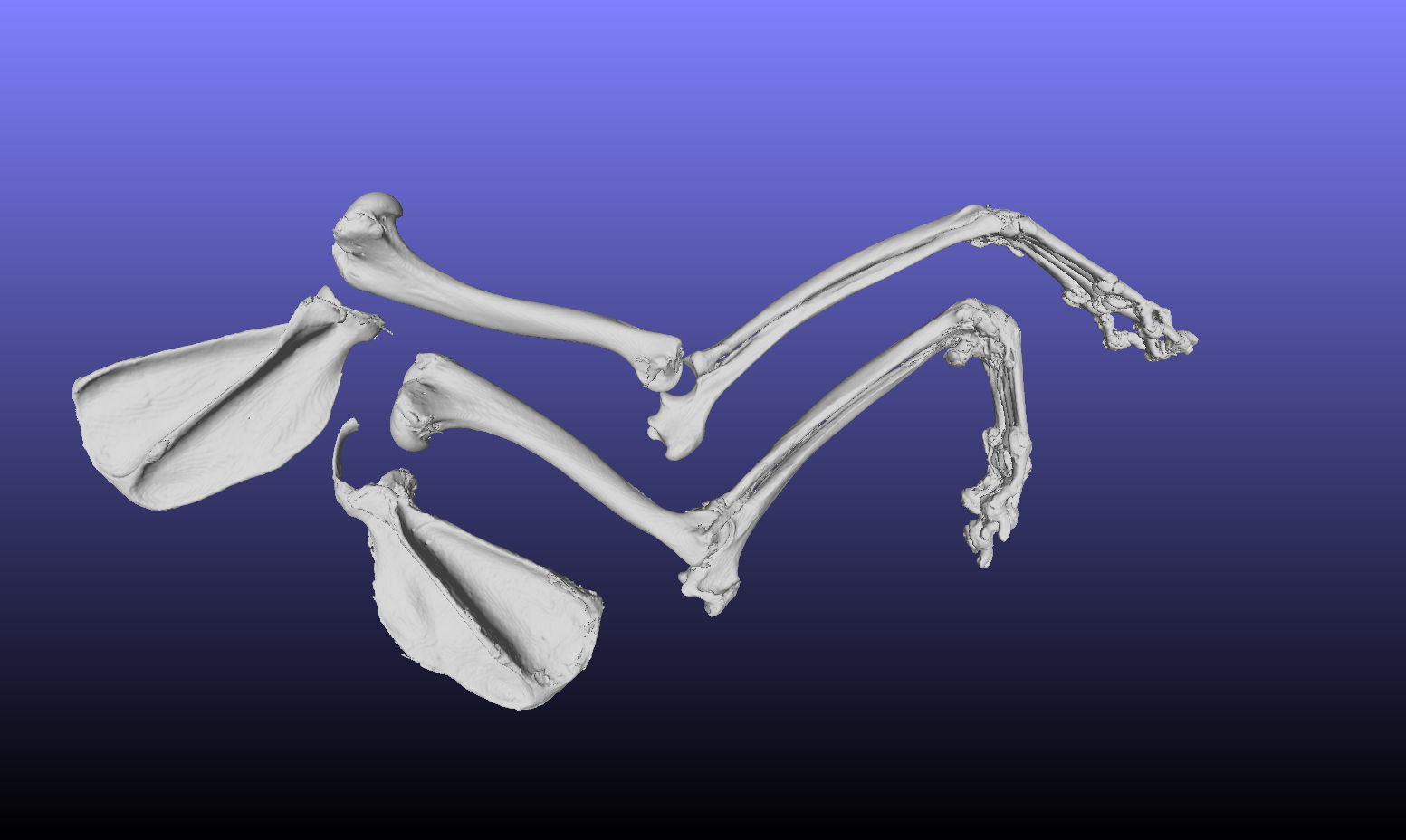

Supplement: Supplemental Information 2 — Specimen was scanned with medical Siemens Somatom Force CT scanner (dual source). The full STL and OBJ files of the CT scans are published online in the repository MorphoSource: https://www.morphosource.org/Detail/SpecimenDetail/Show/specimen_id/32229 [file peerj-08-9866-s002.png]
